# Effect of the 2018 European drought on methane and carbon dioxide exchange of northern mire ecosystems

**DOI:** 10.1098/rstb.2019.0517

**Published:** 2020-09-07

**Authors:** J. Rinne, J.-P. Tuovinen, L. Klemedtsson, M. Aurela, J. Holst, A. Lohila, P. Weslien, P. Vestin, P. Łakomiec, M. Peichl, E.-S. Tuittila, L. Heiskanen, T. Laurila, X. Li, P. Alekseychik, I. Mammarella, L. Ström, P. Crill, M. B. Nilsson

**Affiliations:** 1 Department of Physical Geography and Ecosystem Science, Lund University, Sweden; 2 Climate System Research, Finnish Meteorological Institute, Helsinki, Finland; 3 Department of Earth Sciences, University of Gothenburg, Sweden; 4 INAR Institute for Atmospheric and Earth System Research/Physics, Faculty of Science, University of Helsinki, Finland; 5 Department of Forest Ecology and Management, Swedish Agricultural University, Umeå, Sweden; 6 School of Forest Sciences, University of Eastern Finland, Joensuu, Finland; 7 Bioeconomy and Environment, Natural Resources Institute Finland, Helsinki, Finland; 8 Department of Geological Sciences and Bolin Centre for Climate Research, Stockholm University, Sweden

**Keywords:** greenhouse gas, greenhouse warming potential, wetland, peat, water table

## Abstract

We analysed the effect of the 2018 European drought on greenhouse gas (GHG) exchange of five North European mire ecosystems. The low precipitation and high summer temperatures in Fennoscandia led to a lowered water table in the majority of these mires. This lowered both carbon dioxide (CO_2_) uptake and methane (CH_4_) emission during 2018, turning three out of the five mires from CO_2_ sinks to sources. The calculated radiative forcing showed that the drought-induced changes in GHG fluxes first resulted in a cooling effect lasting 15–50 years, due to the lowered CH_4_ emission, which was followed by warming due to the lower CO_2_ uptake.

This article is part of the theme issue ‘Impacts of the 2018 severe drought and heatwave in Europe: from site to continental scale’.

## 1. Introduction

During the summer of 2018, Northwestern Europe experienced an exceptional drought and heatwave, also affecting Fennoscandian mire ecosystems [1–3]. The drought and associated warm temperatures can alter the short-term hydrological status of mire ecosystems, leading to alterations in biogeochemical processes within these ecosystems. These changes can have a drastic effect on greenhouse gas (GHG) exchange between the mires and the atmosphere [4].

Northern mire ecosystems are characterized by two considerable GHG fluxes, viz. carbon dioxide (CO_2_) uptake and methane (CH_4_) emission, that generate opposite radiative forcing (RF) [5]. On longer timescales, e.g. over millennia, carbon uptake and storage as peat, i.e. sequestration of CO_2_ from the atmosphere, results in a climate cooling effect. Methane emission, on the other hand, has an intense short-term warming effect on the atmospheric radiative balance [6].

The seasonal variation in the CO_2_ and CH_4_ fluxes between the atmosphere and mires has generally been observed to be related to temperature and water table position [7–11]. Dry conditions and lowered water tables hinder CO_2_ uptake [7,12], but they also lead to a reduction in CH_4_ emission [4,13,14]. Thus, the same environmental forcing of GHG exchange of mires can lead to counteracting climatic effects.

To assess the climatic impact of weather events through ecosystem GHG exchange, the differing radiative properties and atmospheric lifetimes of GHGs need to be accounted for. Global warming potential (GWP) is a commonly used metric that integrates the radiative forcing due to a GHG pulse emission over a prescribed time (typically 20 or 100 years) and is expressed as CO_2_ equivalents, i.e. the cumulative RF relative to that of CO_2_ [15]. A more dynamic approach to compare the effects of different GHG fluxes is to examine the development of instantaneous RF due to these fluxes [16,17].

In addition to effective metrics, reliable data on ecosystem GHG exchange are needed to assess the climatic impact of weather events. In Sweden and Finland, GHG fluxes are measured at several mire ecosystems using eddy covariance (EC), mostly within national networks of the Integrated Carbon Observation System (ICOS-Sweden and ICOS-Finland). Appropriate environmental parameters are also measured at each site. In this paper, we will use EC and auxiliary data from five sites to analyse the effect of the 2018 European drought on the CO_2_ and CH_4_ fluxes of mire ecosystems in relation to changes in key environmental drivers. Furthermore, we will analyse the climatic effect of the drought-induced changes in GHG fluxes by using both the GWP and dynamic RF approaches.

## 2. Material and methods

**Figure 1.**
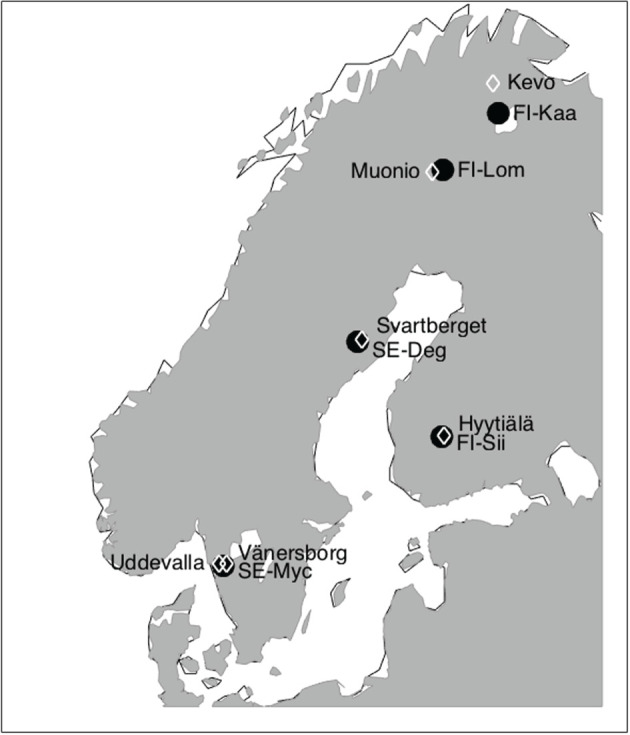
Locations of the mire flux measurement sites used in this study (black dots). FI-Kaa: Kaamanen; FI-Lom: Lompolojänkkä; FI-Sii: Siikaneva; SE-Myc: Mycklemossen; SE-Deg: Degerö (table 1). Also indicated are the weather stations providing long-term climate data listed in table 3 (white diamonds).

We selected natural mire ecosystems that have EC measurements of both CO_2_ and CH_4_ fluxes during 2018 and at least one additional reference year of data. The sites are listed in table 1 and their locations are shown in figure 1. Many of these stations are either ICOS stations or in the process of becoming such and thus the measurements follow the ICOS protocols of CO_2_ and CH_4_ fluxes, and those of auxiliary parameters [24–27]. The average temperature at the sites ranges from −1.4°C to +6.8°C. None of these sites contain permafrost. The vegetation at the mires is listed in table 2, with associations to different mire types.

**Table 1.** Flux sites and their climate conditions.

site	location	type	pH	references
Degerö	64°11′ N 19°33′ E 270 m.a.s.l.	oligotrophic fen	3.9–4.0	[18–20]
Kaamanen	69°08′ N 27°16′ E 155 m.a.s.l.	meso-eutrophic fen	3.7–5.5	[21,22]
Lompolojänkkä	68°00′ N 24°13′ E	mesotrophic fen	5.5–6.5	[23]
Mycklemossen	58°21′ N 12°10′ E	oligotrophic fen with bog characteristics	3.9–4.0	
Siikaneva	61°50′ N 24°12′ E 162 m.a.s.l.	oligotrophic fen	3.2–3.9	[8,10]

**Table 2.** Dominating vascular plant vegetation on the five mire sites (1 = presence of the species). Mire type indicates the species main distribution range according to the Northern vegetation classification by Påhlsson [28]; nutrient poor ombrotrophic bog (B) and minerotrophic fens in order of increasing nutrient availability: poor fen (PF), intermediate fen (IF) and moderate fen (MF). G indicates that the species can be found in all four mire types, and if present in several types the preferred mire type is indicated by *.

species	mire type	Mycklemossen	Degerö	Siikaneva	Kaamanen	Lompolojänkkä
*Calluna vulgaris*	B	1				
*Erica tetralix*	B	1				
*Empetrum nigrum*	B				1	
*Ledum palustre*	B				1	
*Vaccinium uliginosum*	B				1	
*Vaccinium vitis-idaea*	B				1	
*Rubus chamaemorus*	B, PF		1	1	1	
*Eriophorum vaginatum*	B*, PF	1	1	1		
*Rhynchospora alba*	B*, PF	1				
*Carex lasiocarpa*	PF, IF, MF			1		1
*Carex rostrata*	PF, IF, MF			1	1	1
*Eriophorum angustifolium*	PF, IF, MF				1	
*Carex chordorrhiza*	PF, IF*, MF				1	1
*Carex aquatilis*	IF, MF				1	
*Carex livida*	IF, MF				1	
*Carex magellanica*	IF, MF					1
*Carex buxbaumii*	MF				1	
*Andromeda polifolia*	G		1	1		1
*Vaccinium oxycoccus*	G		1	1		1
*Carex limosa*	G		1	1	1	
*Trichophorum cespitosum*	G		1			
Plant community composition taken from		Ström unpubl. results	[18,19]	[8]	[21]	[23]

The effect of drought on GHG fluxes was estimated as differences in the cumulative annual CO_2_ and CH_4_ fluxes between 2018 and a reference period (ΔF_CO2_ and ΔF_CH4_). The reference period was selected as a single year or several years with rainfall and temperature close to the 30-year average (tables 3 and 4). However, flux data availability places a strong constraint on this. For some sites, only a few years of data exist on both CO_2_ and CH_4_ fluxes, and the maximum length of time series for any of the sites was 15 years. As a result of flux data availability, these reference years vary among different mire sites and the environmental conditions during these years may slightly deviate from the long-term average climatological conditions. We related the changes in annual cumulative fluxes to average changes in temperature and water table in summertime, as the drought and heatwave were most conspicuous during this period. The significances of these relations were estimated by non-parametric Spearman's rank correlation test (Matlab Matlab R2015b, corr function). We also compared the apparent temperature dependence of methane emission during the drought and reference years using bin-averaged daily mean methane fluxes. For this, we used daily mean peat temperatures and 2°C bins starting at 0°C.

**Table 3.** Overview of climate datasets from weather stations. For Utsjoki Kevo and Muonio, the reference year is 2017. For Vindeln Svartberget, the reference year is the average of 2015–2016. For Juupajoki Hyytiälä, the reference year is the average of 2010–2013. For Vänersborg and Uddevalla, the reference year is 2016.

station (mire)	location	source	mean annual precipitation [mm]			mean annual temperature [°C]		
			1981–2010	ref.	2018	1981–2010	ref.	2018
Utsjoki Kevo (Kaamanen)	69°43′ N 27°01′ E	FMI	433	519	410	−1.3	−1.1	−0.3
Muonio Alamuonio & kk (Lompolojänkkä)	67°58′ N 23°41′ E	FMI	528	443	472	−0.4	0.3	1.4
Vindeln Svartberget (Degerö)	64°14′ N 19°36′ E	SLU	613	648	546	1.9	3.1	2.8
Juupajoki Hyytiälä (Siikaneva)	61°51′ N 24°17′ E	FMI	703	731	540	3.5	4.3	4.8
Vänersborg (Mycklemossen)	58°21′ N 12°22′ E	SMHI	803	655	599	6.8	7.7	8.2
Uddevalla (Mycklemossen)	58°22′ N, 11°56′ E	SMHI	990	886	820	n.a.	n.a.	n.a.

**Table 4.** Annual carbon dioxide and methane fluxes, and the corresponding GWP-based CO_2_ equivalents of the difference between 2018 and the reference year (ΔCO_2_-eq). Global warming potentials of CH_4_ [15]: GWP_20_ = 84, GWP_100_ = 28.

	CO_2_ reference g C m^−2^	CO_2_ 2018 g C m^−2^	CH_4_ reference g C m^−2^	CH_4_ 2018 g C m^−2^	ΔCO_2_-eq 20 yr	ΔCO_2_-eq 100 yr
Degerö	−31.4 (2015–2016)	15.2	11.4 (2015–2016)	9.5	−36	100
Kaamanen	−8.5 (2017)	−5.6	7.6 (2017)	6.8	−80	−20
Lompolojänkkä	−29.1 (2017)	−56.0	15.0 (2017)	22.0	680	160
Mycklemossen	−1.4 (2016)	54.7	9.7 (2016)	5.6	−260	51
Siikaneva	−78.8 (2010–2013)	18.4	11.5 (2010–2013)	7.6	−74	220

For the long-term climate reference, we used the 1981–2010 monthly precipitation and monthly average air temperature data from nearby weather stations of the Swedish Meteorological and Hydrological Institute (SMHI)^1^ and the Finnish Meteorological Institute (FMI).^2^ For Siikaneva and Lompolojänkkä, we selected the nearby stations of Juupajoki Hyytiälä and Muonio (Alamuonio and kk), respectively. For Degerö, we used climate data collected by the Swedish Agricultural University (SLU) at Vindeln Svartberget. For Mycklemossen, we used precipitation from Uddevalla and temperature from Vänersborg, and for Kaamanen we used Utsjoki Kevo. An overview of these data sets is given in table 3.

Annual CO_2_ and CH_4_ flux time series were derived from the half-hourly EC flux data. Missing observations due to atmospheric conditions not fulfilling micrometeorological flux quality criteria or due to instrument malfunctions were filled in the time series. The CO_2_ fluxes were gap filled as in Wutzler *et al.* [29]. For CH_4_ fluxes, daily averages were calculated [10] and gap filling was conducted by linear interpolation. The uncertainty caused by linear interpolation was assessed by creating artificial gaps in data, representing the number and distribution of gaps in original data, and interpolating the resulting time series. Repeating this 100 times indicated uncertainty generally below 10%. As the automatic water table measurement at the Kaamanen site was not operational in 2017–2018, we used averages of manual measurements to calculate the monthly water table depths at this site.

The statistical significance of difference in the daily CO_2_ and CH_4_ fluxes between 2018 and the reference years was tested using the non-parametric Mann–Whitney–Wilcoxon test (Matlab R2015b, ranksum function, 5% significance level). The test was conducted for July–August, which was the peak flux period and had a large 2018-to-reference difference in water table at most sites.

To compare the climatic effects of the drought-induced changes in CO_2_ and CH_4_ fluxes, we used the GWPs of CH_4_ with two different time horizons, 20 and 100 years, referred to as GWP_20_ (=84) and GWP_100_ (=28). Multiplying the change in the annual CH_4_ mass flux by these GWP values results in fluxes in which CO_2_ and CH_4_ are expressed in common units, i.e. CO_2_ equivalents [15]. To characterize the dynamics of the radiative effect of the GHG flux changes in more detail, we calculated the radiative forcing due to these changes, i.e. again adopting a ‘normal’ year as a reference. We used the impulse-response model described by Lohila *et al*. [16] and subsequently updated to include the indirect RF due to atmospheric CH_4_-to-CO_2_ oxidation [30], revised radiative efficiencies [31] and future GHG concentration scenarios [32]. The use of this method allowed us to estimate the decline of an atmospheric GHG perturbation and the related instantaneous RF over time. We also positioned the mires to the instantaneous RF switchover time diagram based on the ratio of changes in annual CH_4_ and CO_2_ fluxes [6].

## 3. Results

**Figure 2.**
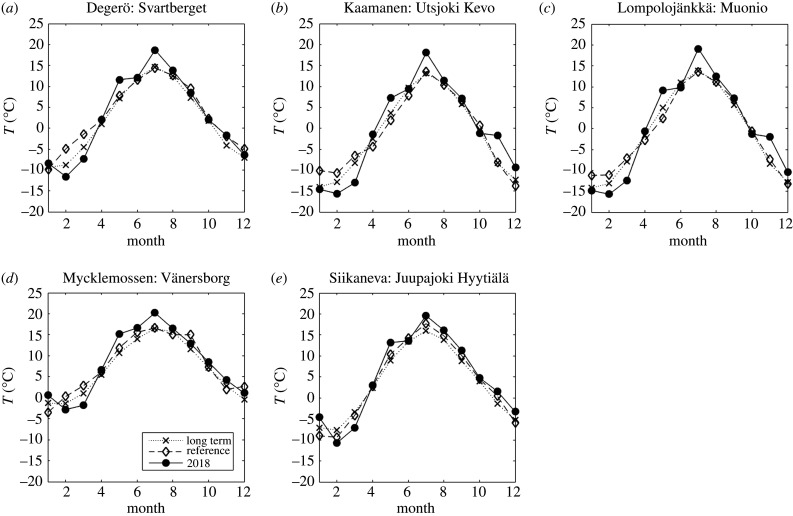
Annual cycle of monthly average air temperatures: long-term mean (crosses); reference period (diamonds); 2018 (dots) from weather stations listed in table 3.

**Figure 3.**
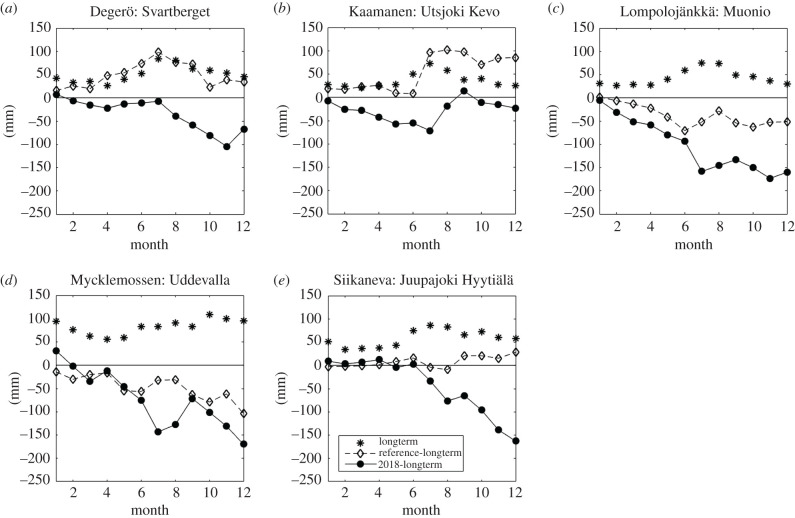
Long-term annual cycle of monthly precipitation (asterisks); cumulative difference of monthly precipitation from long-term average during reference period (diamonds) and 2018 (dots). Data from weather stations listed in table 3.

**Figure 4.**
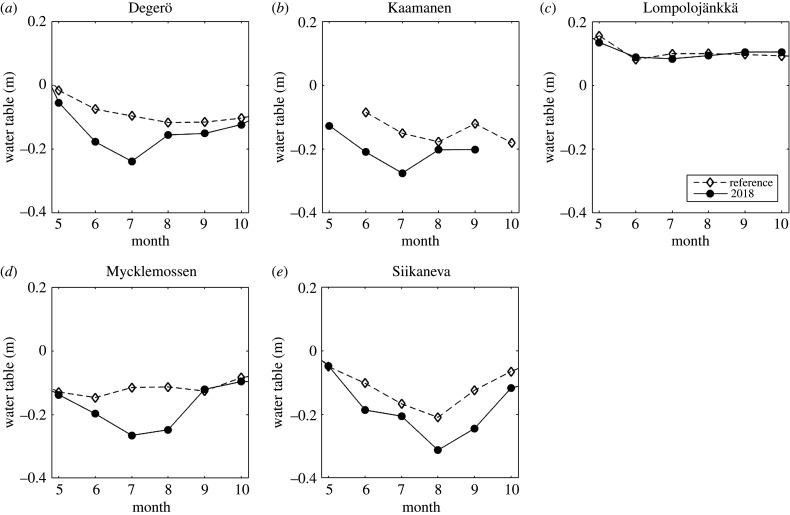
Summertime water table position during the reference period (diamonds) and 2018 (dots).

The summer of 2018 was warmer than on average during the 30-year period of 1981–2010 at all weather stations near the flux measurement sites, with May and July being especially warm (table 3 and figure 2). Temperatures in the selected reference years were close to the 30-year averages during the summer periods at the three northernmost sites, while at the two southernmost sites, the reference summertime temperatures were somewhat higher than the 30-year mean. In 2018, annual precipitation was considerably lower than the 30-year mean, especially at Mycklemossen/Uddevalla and Lompolojänkkä/Muonio, where the drought conditions prevailed during the whole year (figure 3). It is noteworthy that at Mycklemossen/Uddevalla the years 2016, 2017 and 2018 all had below-normal annual precipitation. At Siikaneva/Juupajoki and Degerö/Svartberget, the precipitation in the first half of 2018 was close to the 1981–2010 average, but the end of the year was much drier. At Utsjoki Kevo, the annual precipitation in 2018 was close to the long-term average but the first half of the year had below-average precipitation. The water table at all mires, except for Lompolojänkkä, was lower in summer 2018 than during the reference years (figure 4).

Thus, all mire sites except for Lompolojänkkä experienced a dry year in 2018, as judged from the variations of the water table position. The differences in precipitation between 2018 and reference years at different mires did not correlate with the corresponding differences in water table position.

**Figure 5.**
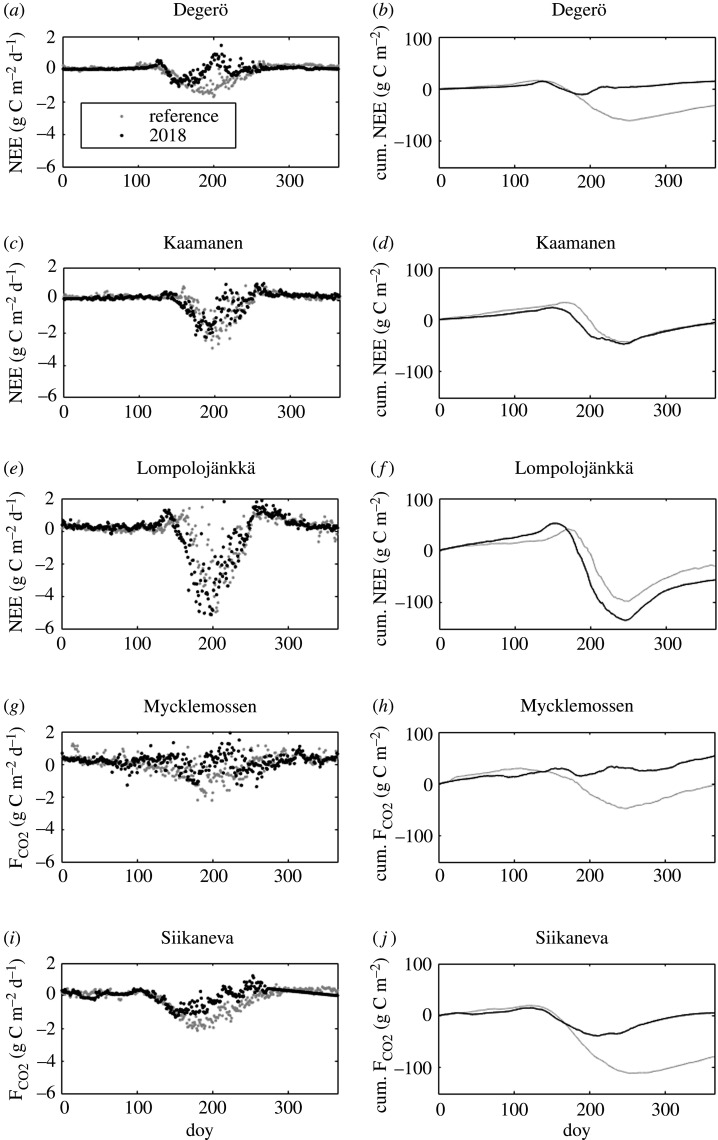
Daily (left panels) and cumulative net (right panels) CO_2_ fluxes during the reference period (grey dots and line) and 2018 (black dots and line). doy, day of year.

**Figure 6.**
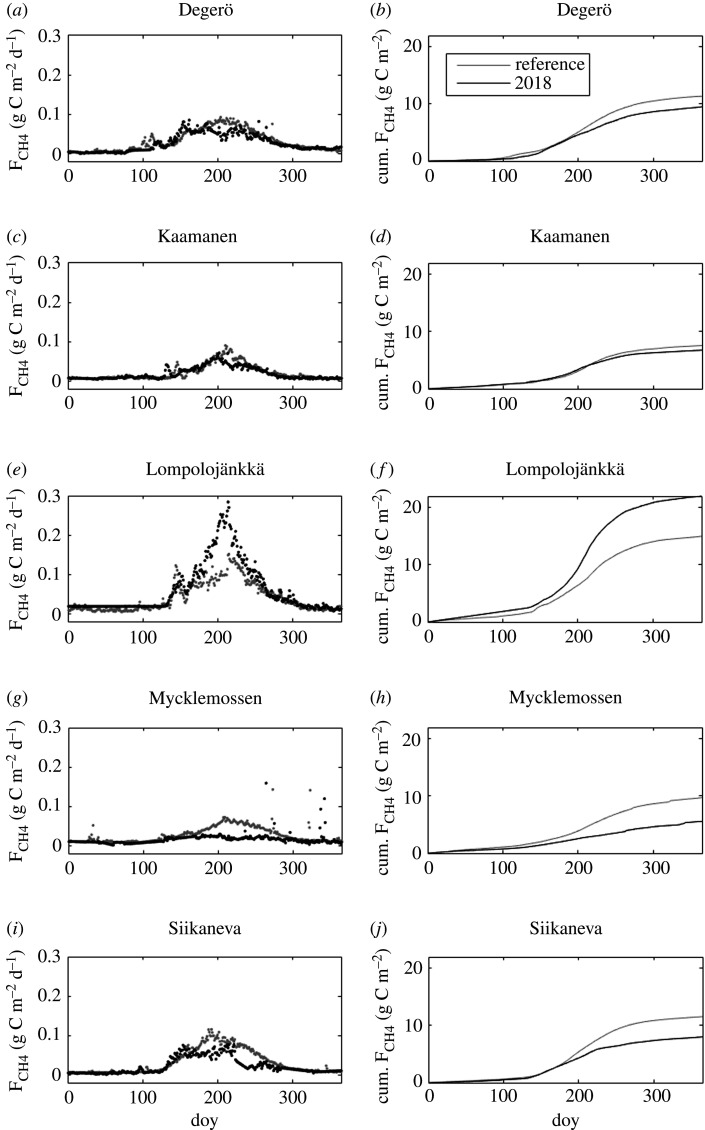
Daily (left panels) and cumulative (right panels) CH_4_ emission during the reference period (grey dots and line) and 2018 (black dots and line). doy, day of year.

All the sites showed a typical annual cycle of both daily CO_2_ and CH_4_ fluxes, with CO_2_ uptake in summer and release outside the growing season, and CH_4_ emission peaking during summer months (figures 5 and 6). The effects of drought and heatwave on CO_2_ exchange is conspicuous, with reduced summertime CO_2_ uptake at Degerö, Mycklemossen and Siikaneva, and increased uptake at Lompolojänkkä. Summertime CH_4_ emission is reduced at all sites except Lompolojänkkä. The difference in daily CH_4_ fluxes during July–August between 2018 and the reference period was significant for all sites. For CO_2_ fluxes, the difference was significant for all sites except Lompolojänkkä.

The cumulative CO_2_ fluxes at Degerö, Lompolojänkkä and Siikaneva showed annual CO_2_ uptake in the reference years, whereas at Mycklemossen and Kaamanen the cumulative net CO_2_ uptake was close to zero (figure 5). In 2018, annual CO_2_ uptake was reduced at all sites except for Lompolojänkkä and three sites acted as CO_2_ sources at an annual timescale. The annual cumulative ecosystem CH_4_ emission was reduced during 2018 as compared to the reference years, except at Lompolojänkkä (figure 6). Not accounting for this site, the change in the annual CO_2_ flux (ΔF_CO2_) ranged from 3 g C m^−2^ to 100 g C m^−2^, while the change in annual CH_4_ flux (ΔF_CH4_) ranged from −0.8 g C m^−2^ to −4 g CH_4_ m^−2^. Lompolojänkkä had opposite changes compared to the other sites, with increased CO_2_ uptake (ΔF_CO2_ = −27 g C m^−2^) and CH_4_ emission (ΔF_CH4_ = 7.0 g C m^−2^).

**Figure 7.**
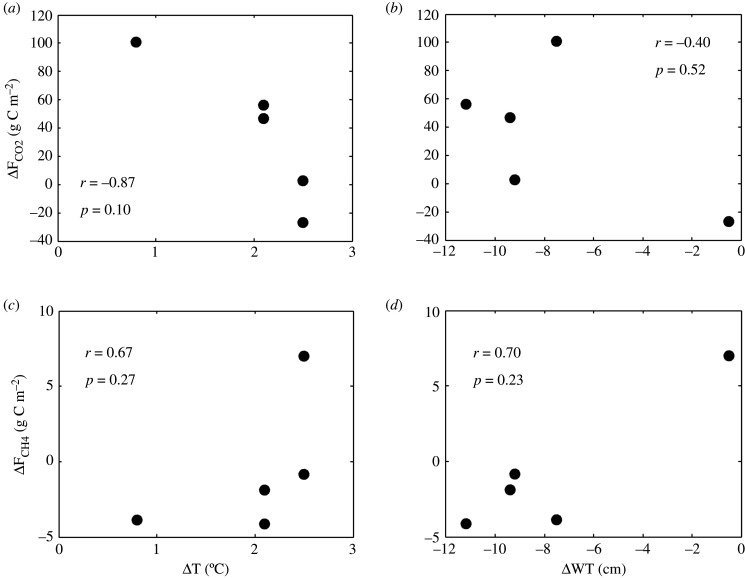
Relationship between changes in annual GHG fluxes (ΔF_CO2_ and ΔF_CH4_) and the summertime average changes in air temperature (ΔT; left panels) and summertime average change in water table position (ΔWT; right panels). Correlation and p-value are according to non-parametric Spearman's rank correlation.

**Figure 8.**
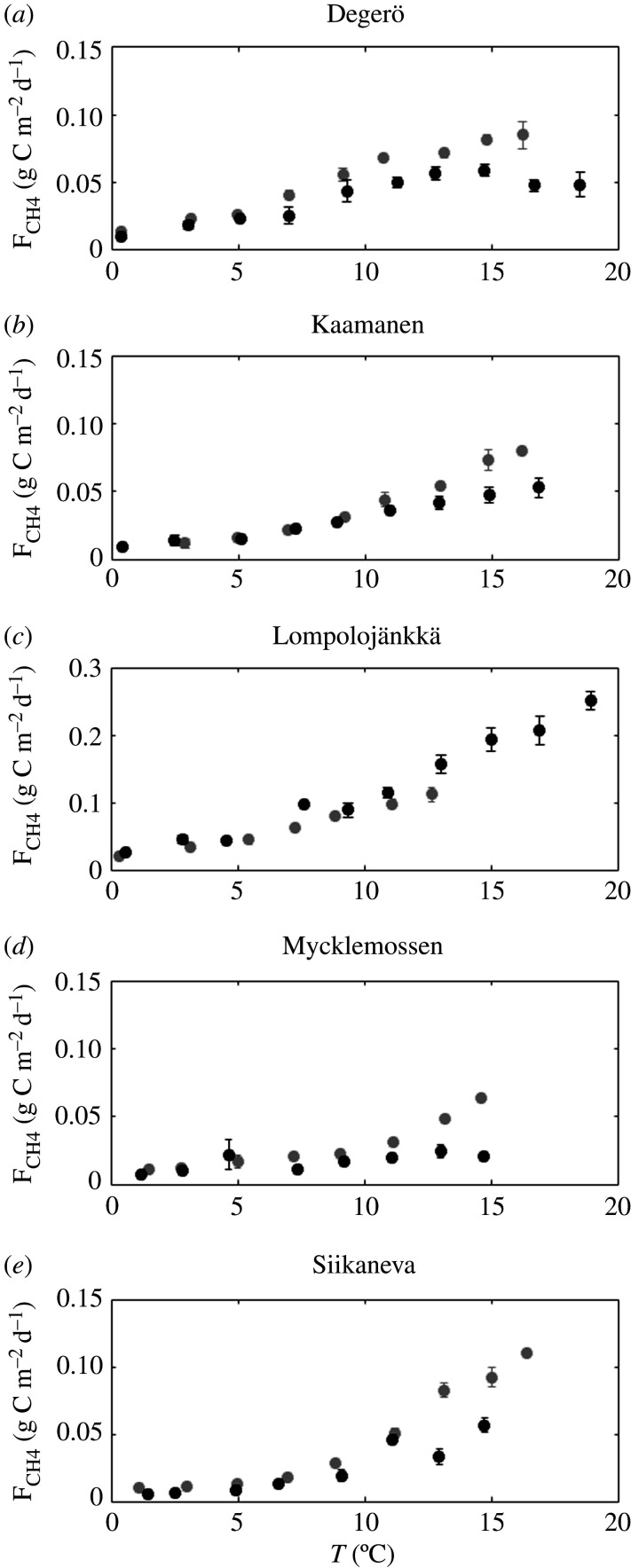
Bin-averaged CH_4_ emission against peat temperature in the reference perion (grey dots) and 2018 (black dots). Error bars correspond to 1.96 times the standard error of the mean.

The relations of ΔF_CH4_ and ΔF_CO2_ to the 2018-to-reference difference in summertime water table position (ΔWT) or the difference in air temperature were not significant (ΔT) (figure 7). However, at all sites with a lowered water table, the CH_4_ emissions at a given temperature bin were generally lower during the drought year than in the reference years (figure 8).

For all mires with a substantial water table lowering in 2018, the drought-induced changes in the annual CO_2_ and CH_4_ balances, estimated above, correspond to a cooling effect when the fluxes are expressed as GWP_20_-based CO_2_ equivalents (negative CO_2_ equivalents, table 4). This indicates the short-term dominance of reduced CH_4_ emissions. However, the corresponding GWP_100_-based values were positive, indicating warming, at all sites except for Kaamanen.

**Figure 9.**
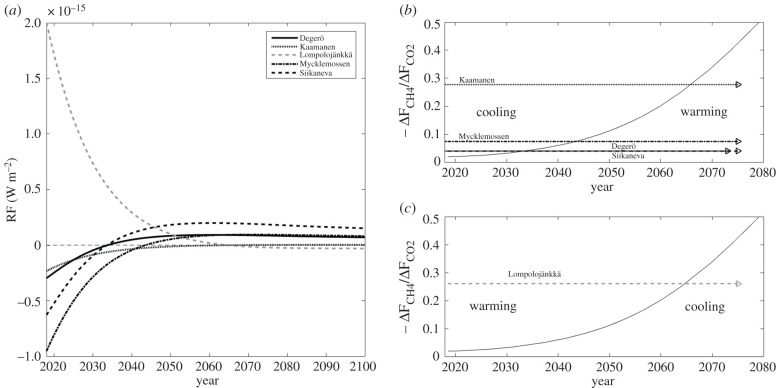
(*a*) Time evolution of instantaneous radiative forcing (RF) due to the change in net CO_2_ uptake (ΔF_CO2_) and CH_4_ emission (ΔF_CH4_) during 2018, as compared to the reference period. (*b*,*c*) The dependence of timing of the RF switchover time (sign change) on the ΔF_CO2_/ΔF_CH4_ ratio for sites with a negative ΔF_CH4_ and a positive ΔF_CO2_ (*b*) and a positive ΔF_CH4_ and a negative ΔF_CO2_ (*c*). The arrows indicate the ΔF_CO2_/ΔF_CH4_ ratio for each site and the corresponding cooling and warming periods.

The instantaneous RF due to GHG flux changes, caused by dry conditions, show an initial cooling effect resulting from the reduced CH_4_ emission at all sites except for Lompolojänkkä (figure 9*a*). Later, the effect of reduced CO_2_ uptake will dominate, causing a warming effect at these sites. Lompolojänkkä, with opposite changes in GHG fluxes as compared to other mires, shows an initial warming and a subsequent cooling effect. The switchover of the instantaneous RF from cooling to warming takes place 15–50 years after the drought year for the mires which experienced a water table drawdown (figure 9*b*), while at Lompolojänkkä the transition was from warming to cooling (figure 9*c*).

## 4. Discussion

The 2018 drought caused a widespread water table drawdown across North European mire ecosystems. This is a reflection of the dry and warm conditions during summer of 2018. In Sweden, precipitation deficit was observed in nearly the whole country from May to July, with Southern Sweden experiencing the highest deficit [1]. Furthermore, May and July were much warmer than the long-term average for the whole of Sweden, while June was somewhat warmer in the south and colder in the north [1]. In Finland, the early summer precipitation deficit was more pronounced in the southern part of the country [2], with warmer than average summer for the whole county [3]. However, local hydrological features related to e.g. topographical position can cause some mires to be less sensitive to climatic variations, as seen at the Lompolojänkkä mire.

We observed similarities in the change of annual GHG fluxes at all the mires with a water table drawdown, with a reduction of both CO_2_ uptake and CH_4_ emission. Three out of the four mires with lowered water table turned from CO_2_ sinks to sources during 2018. The reduction of CH_4_ emission was more moderate, the change being mostly less than 20% of the emission during the reference year, with the exception of Mycklemossen (42%). Mycklemossen is the southernmost of the mire sites in this study and is located within the area most affected by the 2018 drought. It also experienced drier than average conditions in 2016 and 2017, the effects of which may have carried over to 2018. Furthermore, Mycklemossen has most ombrotrophic bog characteristics while the other mires show more minerogenic fen characteristics (table 2). As bogs typically have a lower water table as compared to minerogenic fens and have a lesser coverage of aerenchymatous plants, their CH_4_ emission may be more sensitive to dry conditions.

We could not establish statistically significant correlations between the changes of annual CO_2_ exchange and annual CH_4_ emission with summertime temperature or water table level. However, the apparent dependence of CH_4_ emission on peat temperature shows a clear 2018-to-reference difference in all mires with a lowered water table. Similar differences in the apparent temperature dependence of CH_4_ emission have also been observed previously [33,34]. At Lompolojänkkä, where the water table was not drawn down, the temperature response of CH_4_ emission was similar in 2018 and the reference year. The high peat temperature at Lompolojänkkä in 2018 can explain the very high CH_4_ emission in that year. On the other hand, at Degerö, which also had relatively high peat temperatures in 2018, the CH_4_ emission was clearly lowered due to the lower water table. Thus, it seems obvious that both the water table level and peat temperature play a role in this variation. The use of such dependencies e.g. for upscaling the climatic effects of droughts would additionally require establishing a relationship between water table and precipitation, and peat temperature and air temperature, as water table position and peat temperatures are not parameters commonly measured by weather observation networks.

As the CH_4_ emissions were reduced, this change first dominated the radiative forcing effects over the reduction in CO_2_ uptake and resulted in a temporary cooling effect. According to our RF analysis, this cooling was in most cases limited to the first 15–50 years after the drought year. The length of this period depends on the ratio of the changes in the two GHG fluxes, while the strength of the cooling and warming effects depend on the magnitude of these changes. At Siikaneva and Degerö, with a small reduction in CH_4_ emission as compared to a reduction in CO_2_ uptake, the cooling period is short, whereas for Kaamanen, with a small change in CO_2_ uptake, cooling lasts longer. Mires with a large change in CH_4_ fluxes showed a large initial change in the instantaneous RF. Siikaneva, with the largest reduction in CO_2_ uptake, showed the largest warming after the switchover from cooling to warming. The GWP_20_- and GWP_100_-based metrics, which essentially represent RF integrals, reflect the RF-based analysis.

The short-term climatic effect as shown by both the GWP and RF approaches is very sensitive to the changes in CH_4_ fluxes. As the variation in annual CH_4_ emissions from northern mires can be *ca*. 2 g C m^−2^ [10], the selection of reference years can have a large effect on the estimated short-term climatic forcing, which is affected more by CH_4_ than CO_2_. Ideally, we should compare the CH_4_ emissions during a drought year to a long-term average. Currently, however, very few CH_4_ emission time series exceed 10 years. Thus, the development of long-term flux measurement networks, such as ICOS, is expected to lead to more representative datasets and improved understanding of these climate feedbacks.

## 5. Conclusion

The dry conditions in Northwestern Europe in 2018 led to a lowering of water table position at most, but not all, flux measurement sites on mire ecosystems. The lowered water table led to a reduction of both summertime CO_2_ uptake and CH_4_ emission, and annual exchange of these GHGs. The apparent temperature dependence of CH_4_ fluxes was clearly affected by the lowered water table, but also temperature effects were obvious. Due to the different atmospheric residence times of these GHGs, the cooling effect due to the reduction of CH_4_ emission dominates for the first 15–50 years after the drought, after which the warming effect by the reduced CO_2_ uptake will dominate.

## Endnotes

1 https://www.smhi.se/data/meteorologi/

2 https://en.ilmatieteenlaitos.fi/download-observations#!/

## Data accessibility

The data from Degerö and Lompolojänkkä is available through ICOS Carbon Portal (https://www.icos-cp.eu/). Siikaneva data is available through Avaa portal (https://avaa.tdata.fi/web/smart/smear/search). Mycklemossen data is available through SITES portal (https://data.fieldsites.se/portal/). Data from Kaamanen is available at zenodo (https://zenodo.org/record/3975733). The climate data is publicly available from SMHI and FMI web portals (https://www.smhi.se/data/meteorologi/; https://en.ilmatieteenlaitos.fi/download-observations#!/).
